# Myogenic Cell Transplantation in Genetic and Acquired Diseases of Skeletal Muscle

**DOI:** 10.3389/fgene.2021.702547

**Published:** 2021-08-02

**Authors:** Olivier Boyer, Gillian Butler-Browne, Hector Chinoy, Giulio Cossu, Francesco Galli, James B. Lilleker, Alessandro Magli, Vincent Mouly, Rita C. R. Perlingeiro, Stefano C. Previtali, Maurilio Sampaolesi, Hubert Smeets, Verena Schoewel-Wolf, Simone Spuler, Yvan Torrente, Florence Van Tienen, Study Group, H. Aldearee

**Affiliations:** Division of Cell Matrix Biology and Regenerative Medicine, The University of Manchester, United Kingdom; Department of Immunology & Biotherapy, Rouen University Hospital, Normandy University, Inserm U1234, Rouen, France; Division of Cell Matrix Biology and Regenerative Medicine, The University of Manchester, United Kingdom; Department of Digestive Surgery, Rouen Univesrity Hospital, Rouen, France; Translational Cardiomyology Laboratory, Department of Development and Regeneration, KU Leuven, Leuven, Belgium; Unit of Neurology, Stem Cell Laboratory, Department of Patophysiology and Transplantation, Centro Dino Ferrari, Università degli Studi di Milano, Fondazione IRCCS Cà Granda Ospedale Maggiore, Policlinico, Milan, Italy; Muscle Research Unit, Experimental and Clinical Research Center, a Cooperation between Max-Delbruck-Center for Molecular Medicine in the Helmholtz Association and the Charitè Universitadmedizin Berlin, Berlin, Germany; Translational Cardiomyology Laboratory, Department of Development and Regeneration, KU Leuven, Leuven, Belgium; Department of Immunology & Biotherapy, Rouen University Hospital, Normandy University, Inserm U1234, Rouen, France; Division of Cell Matrix Biology and Regenerative Medicine, The University of Manchester, United Kingdom; Sorbonne Université, Inserm, Institut de Myologie, Centre de Recherche en Myologie, Paris, France; INSpe and Division of Neuroscience, IRCCS Ospedale San Raffaele, Milan, Italy; INSpe and Division of Neuroscience, IRCCS Ospedale San Raffaele, Milan, Italy; Unit of Neurology, Stem Cell Laboratory, Department of Patophysiology and Transplantation, Centro Dino Ferrari, Università degli Studi di Milano, Fondazione IRCCS Cà Granda Ospedale Maggiore, Policlinico, Milan, Italy; Translational Cardiomyology Laboratory, Department of Development and Regeneration KU Leuven, Leuven, Belgium; INSpe and Division of Neuroscience, IRCCS Ospedale San Raffaele, Milan, Italy; ^1^Department of Immunology & Biotherapy, Rouen University Hospital, Normandy University, Inserm U1234, Rouen, France; ^2^Sorbonne Université, Inserm, Institut de Myologie, Centre de Recherche en Myologie, Paris, France; ^3^Manchester Centre for Clinical Neurosciences, Manchester Academic Health Science Centre, Salford Royal NHS Foundation Trust, Salford, United Kingdom; ^4^National Institute for Health Research Manchester Biomedical Research Centre, Manchester University NHS Foundation Trust, The University of Manchester, Manchester, United Kingdom; ^5^Division of Cell Matrix Biology & Regenerative Medicine, The University of Manchester, Manchester, United Kingdom; ^6^Muscle Research Unit, Experimental and Clinical Research Center, a Cooperation Between the Max-Delbrück-Center for Molecular Medicine in the Helmholtz Association and the Charité, Universitätsmedizin Berlin, Berlin, Germany; ^7^InSpe and Division of Neuroscience, Istituto di Ricerca e Cura a Carattere Scientifico (IRCCS) Ospedale San Raffaele, Milan, Italy; ^8^Department of Medicine, Lillehei Heart Institute, Stem Cell Institute, University of Minnesota, Minneapolis, MN, United States; ^9^Translational Cardiomyology Laboratory, Department of Development and Regeneration, KU Leuven, Leuven, Belgium; ^10^Human Anatomy Unit, Department of Public Health, Experimental and Forensic Medicine, University of Pavia, Pavia, Italy; ^11^Department of Toxicogenomics, Maastricht University Medical Centre, Maastricht, Netherlands; ^12^School for Mental Health and Neurosciences (MHeNS), Maastricht University, Maastricht, Netherlands; ^13^School for Developmental Biology and Oncology (GROW), Maastricht University, Maastricht, Netherlands; ^14^Unit of Neurology, Stem Cell Laboratory, Department of Pathophysiology and Transplantation, Centro Dino Ferrari, Università degli Studi di Milano, Fondazione Istituto di Ricerca e Cura a Carattere Scientifico (IRCCS) Cà Granda Ospedale Maggiore Policlinico, Milan, Italy

**Keywords:** cell transplantation, muscle stem cells, muscular dystrophies, mitochondrial myopathies, inflammatory myopathies, sphincter incontinence, volumetric muscle loss

## Abstract

This article will review myogenic cell transplantation for congenital and acquired diseases of skeletal muscle. There are already a number of excellent reviews on this topic, but they are mostly focused on a specific disease, muscular dystrophies and in particular Duchenne Muscular Dystrophy. There are also recent reviews on cell transplantation for inflammatory myopathies, volumetric muscle loss (VML) (this usually with biomaterials), sarcopenia and sphincter incontinence, mainly urinary but also fecal. We believe it would be useful at this stage, to compare the same strategy as adopted in all these different diseases, in order to outline similarities and differences in cell source, pre-clinical models, administration route, and outcome measures. This in turn may help to understand which common or disease-specific problems have so far limited clinical success of cell transplantation in this area, especially when compared to other fields, such as epithelial cell transplantation. We also hope that this may be useful to people outside the field to get a comprehensive view in a single review. As for any cell transplantation procedure, the choice between autologous and heterologous cells is dictated by a number of criteria, such as cell availability, possibility of *in vitro* expansion to reach the number required, need for genetic correction for many but not necessarily all muscular dystrophies, and immune reaction, mainly to a heterologous, even if HLA-matched cells and, to a minor extent, to the therapeutic gene product, a possible antigen for the patient. Finally, induced pluripotent stem cell derivatives, that have entered clinical experimentation for other diseases, may in the future offer a bank of immune-privileged cells, available for all patients and after a genetic correction for muscular dystrophies and other myopathies.

## Introduction

Cell and stem cell therapies have been in the clinics for more than a century, starting with blood transfusion, bone marrow transplantation, and epidermal transplantation for large burns. They have saved millions of lives and all are consolidated therapies, since a time when the concept of stem cells did not exist, at least in its modern meaning (Cossu et al., 2018).

However, it was the explosion of stem cell biology around the beginning of this century that fueled work into clinical translation, with premature enthusiasm based on the unproven assumption that they would be a magical tool to heal virtually any yet untreatable disease. Instead, recent history of stem cell therapy has taught us several lessons and provided us with evidence that could have been predicted. Unfortunately, the enthusiasm for this new therapeutic avenue and the pressure from patients, in a long and often desperate wait for a lifesaving or life-changing therapy, forced research toward clinical experimentation. It is, however true that, although pre-clinical work in small and large animal models is indispensable to predict the possible efficacy of a given experimental strategy, the final and definitive answer is only provided by clinical trials; therefore, a difficult equilibrium has to be found between the need to accumulate sufficient evidence and the risk of delaying trials that may be successful.

We know now that stem cells have produced dramatic clinical results only in diseases affecting epithelia, such as epidermis and cornea, and hematopoietic tissues. This is due to several reasons including the constant self-renewal that only occurs in these tissues, their unique physical nature (a liquid suspension or a bi-dimensional sheet) but the most relevant is the possibility of ablating the majority or the totality, for epithelia, of diseased cells so that engraftment of donor cells may easily attain 80–100% of the cells, virtually replacing the large majority of the resident tissue cell population (Cossu et al., 2018). For comparison, intra-arterial transplantation of donor mesoangioblasts in patients with Duchenne muscular dystrophy (DMD) resulted in 0.7% of engraftment in the muscles of the youngest patient, clearly insufficient to detect any clinical benefit, even though donor derived dystrophin was clearly detected (Cossu et al., 2015). In reality other genetic diseases have been successfully treated, such as lysosomal storage diseases, but also in this case the strategy has relied upon the transplantation of HSC genetically corrected to over-express the product of the mutated gene in HSC-derived phagocytes (Staal et al., 2019).

While the advantage of life-long self-renewing stem cells is obvious for the above-mentioned tissues, skeletal muscles are believed to renew only a few times in a life-time under physiological conditions and adjust to increasing demand by hypertrophy rather than by hyperplasia. Maybe this is at least one reason why there are stem/progenitor cells in skeletal muscle with a limited self-renewal capacity, at least when compared with HSC (Rocheteau et al., 2012). The biology of muscle stem cells is now a heavily investigated field, though mainly in rodents, but also increasingly in human cells (Marg et al., 2014, 2019; Snijders et al., 2015); moreover, in muscle diseases with continuous cycles of degeneration/regeneration, such as DMD, regeneration after a while essentially ceases due to the exhaustion of the resident muscle stem cells/satellite cells and the tissue is progressively replaced by fat and connective tissue (Lu et al., 2014). It has also been shown that fibro-adipogenic progenitors play a complex role in regeneration by promoting satellite cell activation even if their fate is to produce fat and fibrotic scar tissue (Joe et al., 2010). In general, dealing with fibrosis is crucial for any specific therapeutic strategy. Whichever the case, one lesson learned from many other genetic diseases is that intervention must be as precocious as possible or hope of efficacy will essentially vanish.

In this review we will start with a brief history of cell transplantation for diseases of skeletal muscle, followed by a description of different types of progenitor cells for transplantation. These include several “non-myogenic” cell types, such as mesenchymal stromal (often referred as “stem”) cells or hematopoietic stem cells, based on the fact that these cells produce growth factors and cytokines thus eliciting some paracrine beneficial effect. Recent history of cell transplantation for heart diseases, not to be reviewed here, has clearly shown that a paracrine effect alone is not sufficient to guarantee significant and long-lasting clinical efficacy.

**FIGURE 1:**
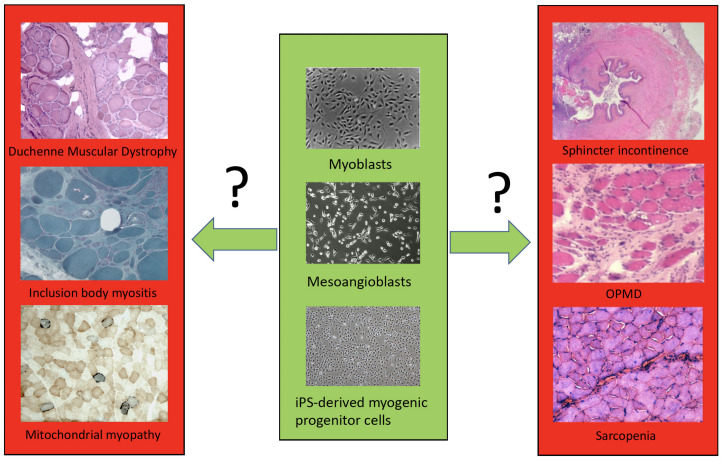
A simplified scheme of the different cell types (only few examples are shown) available for transplantation in different muscle disorders.

We then will examine in detail cell transplantation for DMD, other forms of muscular dystrophies and mitochondrial myopathies, inclusion body myositis, sphincter incontinence, sarcopenia, cachexia and volumetric muscle loss (VML) (Figure 1). By comparing these different muscle diseases, we aim to learn common rules and peculiarities, reasons for failure or incomplete success and set the basis for a new phase of cell therapy which will also capitalize from the impressive new knowledge recently acquired on satellite and other myogenic cells.

## Brief History of Cell Therapy for Muscle

The pioneering work of Partridge et al. (1989) performed in mdx mouse model for DMD confirmed the ability of injected myoblasts to colonize striated muscle and restore function. This stimulated the use of allogeneic myoblasts in clinical trials to restore muscle function in DMD (Huard et al., 1992; Karpati et al., 1993; Tremblay et al., 1993; Mendell et al., 1995). These first trials were followed by other pilot investigations in DMD or Becker muscular dystrophy (BMD) (Gussoni et al., 1997; Miller et al., 1997; Neumeyer et al., 1998). These pioneering studies unfortunately resulted in limited or no clinical effect, mostly because of immunological incompatibility and massive death of transplanted cells, which led to a diffuse skepticism that this strategy may work in the future. However, they established the feasibility of cell therapy in human skeletal muscle and revealed many bottlenecks that were totally unexpected. Many questions were raised and discussed within the scientific community following these early trials, such as local vs. systemic delivery, allografts vs. autologous transfer (and the necessity to correct the implanted cells), the state of the target muscle(s) and the impact of fibrosis, among many others (Negroni et al., 2016). Since then, and in response to these debates, several stem cell candidates with a myogenic potential have been tested, either in animal models, or more rarely (such as mesoangioblasts or CD133 cells) in clinical trials. The rationale behind this was directly derived from the experience gained in the field of gene therapy for congenital immune deficiencies. As long as lymphocytes were genetically corrected, the clinical benefit remained linked to the survival of transduced cells, whereas when long-term self-renewing stem cells were transduced, the clinical benefit appeared to be long-lasting, as genetically corrected lymphocytes were continuously produced and there is today a thirty-year follow up of the first treated patients none of whom has had any relapse of the original disease (Ferrua and Aiuti, 2017). As mentioned above, the characterization of muscle stem/progenitor cells is still ongoing; however, several trials with satellite cells and other progenitors have been carried out and are described in the following paragraphs.

In general, cell therapy to treat muscular dystrophies has been based on a trial-and-error strategy, and while very few recent trials have shown some clinical benefit, each trial has brought a significant load of information that is instrumental to increase future chances of success.

## Different Types of Progenitor Cells for Transplantation

Ideally, progenitors for muscle cell therapy should be endowed with the following properties: (1) high muscle regenerative capacity upon delivery; (2) contribution to the muscle stem cell pool, essential for long-term homeostasis; (3) ability to maintain regenerative properties upon *in vitro* culture, which is necessary for expansion from a small biopsy; (4) amenability for genome engineering; (5) extravasation potential following systemic delivery (for widespread muscle diseases); (6) muscle tropism following delivery, such as few/no cells will be retained by the liver, lung or other organs; (7) elicit low to no immune response, which is particularly relevant for sustained expression of the therapeutic gene over a long period of time; (8) simple and robust manufacturing. Over the past three decades, many studies have attempted to identify cell populations that could be effectively used to promote skeletal muscle repair in a clinical setting (reviewed in Martin et al., 2019; Ortiz-Vitali and Darabi, 2019; Biressi et al., 2020; Łoboda and Dulak, 2020; Sun et al., 2020). Here we provide a brief overview of these efforts and discuss novel technologies that could improve or complement future cell-based interventions. Table 1 shows a synthetic overview of the different cell types.

**TABLE 1:** A simplified scheme of most common cell types used for transplantation, with features, limitations and current use in trials.

Cell type	Main features	Limitations	Tested in clinical trials
Satellite cells	• Endogenous stem cell population that contributes to long term muscle homeostasis • Single cell transplantation studies evidenced a high regenerative capacity	• Stem cell properties are not preserved upon *in vitro* culture • Isolation of a therapeutically relevant number of satellite cells requires a substantial amount of muscle tissue	No
Myoblasts	• Easy isolation and manufacturing following a small muscle biopsy • Banking may allow use of HLA-matched preparations • May be genetically corrected	• Large numbers are required for extensive muscle regeneration • Low survival post-injection	Yes
Mesoangioblasts	• Easy isolation and manufacturing following a small muscle biopsy • Muscle homing upon intra-arterial systemic delivery • Myogenic potential is preserved during *in vitro* expansion and genetic manipulation.	• Large numbers are required for extensive muscle regeneration • Myogenic potency lower than in myoblasts.	Yes
Muscle interstitial populations	• Side population cells are endowed with muscle homing potential • TWIST2+ cells display fiber type selectivity	• Autologous transplantation requires muscle tissue biopsy • Undefined muscle regenerative potential • Undefined cell manufacturing process	No
Mesenchymal stromal cells	• Established isolation process • Potential immunomodulatory function	• Conflicting data in terms of *in vivo* muscle regenerative potential	Yes
CD133+ cells	• Easy isolation and manufacturing following a small muscle biopsy • Muscle homing upon systemic delivery • Myogenic potential is preserved during *in vitro* expansion and genetic manipulation	• Large numbers required for extensive muscle regeneration are difficult to be reached.	Yes
iPSC-derived myogenic progenitors	• Amenable to genome editing prior to specification of the myogenic lineage • Banking may allow use of HLA-matched preparations • Maybe genetically corrected • Myogenic potential is preserved during *in vitro* expansion • Muscle homing upon systemic delivery (demonstrated for murine cells)	• Large numbers are required for extensive muscle regeneration • Potential risk of teratogenicity if residual pluripotent stem cells are present in the myogenic cell preparation	No

### Satellite Cells

Skeletal muscle homeostasis is ensured by a tissue-resident stem cell population referred as satellite cells. These cells are located beneath the sarcolemma and express the transcription factor Pax7 (Mauro, 1961; Seale et al., 2000). In adult healthy muscle, satellite cells are in a quiescent state, characterized by low transcriptional and metabolic activities. In response to various cues, these cells quickly exit quiescence, increase their cellular activities and generate a population of transient amplifying progenitors, referred as myoblasts or myogenic progenitor cells, which ultimately fuse to and repair the damaged myofibers (Scharner and Zammit, 2011). Importantly, each activation cycle is also associated with the replenishment of the muscle stem cell pool, which involves the concerted action of several effectors controlling symmetric and asymmetric satellite cell division (Feige et al., 2018). Various studies have demonstrated that satellite cells are endowed with a high regenerative capacity upon intramuscular injection in murine models. While this property makes satellite cells the obvious candidate population for cell therapy, their clinical use is still problematic. In fact, satellite cells quickly lose their regenerative potential following *in vitro* culture (Montarras et al., 2005; Sacco et al., 2008), thus implying the requirement of a substantial amount of donor muscle tissue to obtain sufficient numbers of these progenitors for downstream applications. Furthermore, satellite cells show no muscle homing ability upon systemic delivery (Choi et al., 2020), which represents a significant limitation for the treatment of pathologies affecting multiple muscle types. Efforts over the past few years have identified critical aspects regulating satellite cell maintenance and quiescence: substrate rigidity, niche components, signaling pathways, and many more (reviewed in Feige et al., 2018). An increasing number of reports are now focusing on the characterization of human satellite cells, which can be prospectively isolated using various surface markers (CD56, CD82, β1-Integrin, EGFR) (Charville et al., 2015; Alexander et al., 2016; Uezumi et al., 2016). Combined to advanced culture systems, these ongoing studies may enable the effective therapeutic application of human satellite cells to treat skeletal muscle degenerative disorders.

### Myoblasts

Myoblasts constitute the committed derivative of satellite cells, which ultimately fuse to and, if necessary, repair the myofiber (Forcina et al., 2020). At the molecular level, myoblasts are defined by the expression of the muscle regulatory factors (MRFs) Myf5 and MyoD, which are induced following satellite cell activation (Buckingham and Rigby, 2014). Myoblast-mediated muscle repair involves cell cycle exit and differentiation into myocytes, processes controlled at the molecular level by two distinct MRFs: Myogenin and Myf6. As myoblasts transit into myocytes, additional key steps are required for myofiber formation, including cell fusion and sarcomere formation, among others (Buckingham and Rigby, 2014; Petrany and Millay, 2019). Although myoblasts display lower regenerative potential compared to satellite cells and limited migration from the injection site, there are advantages, such as easy collection and expansion. Accordingly, myoblast cultures, defined as CD56+ cells, are established by digesting muscle biopsies followed by *in vitro* expansion (Blau and Webster, 1981). Several methods to enrich for “stem cell-like” myogenic progenitors have been reported (Deasy et al., 2001; Hashimoto et al., 2004; Alonso-Martin et al., 2016; Maesner et al., 2016; Hicks et al., 2018). For example, myoblasts, expanded after a period of hypothermia without enzymatic digest give rise to pure myogenic cell populations with high regenerative capacity that repopulate the stem cell niche (Marg et al., 2014). Injection of myoblasts in non-human primate models provided important preclinical data about host-mediated immune rejection, immunosuppression regimen, number of injections, delivery system, and migration from the injection site (Skuk et al., 2011, 2014; Skuk and Tremblay, 2012, 2017, 2020).

### Side Population and Interstitial Muscle Cells

Additional myogenic cell populations have been identified in the skeletal muscle, including side population cells, PW1+ cells and TWIST2+ cells. Originally identified in the hematopoietic system, side population cells express the ABCG2 transporter, which accounts for the dye-exclusion properties exhibited by these cells (Gussoni et al., 1999). Side population cells have been reported to home to skeletal muscles upon systemic delivery but, unfortunately, possess limited regenerative properties (Muskiewicz et al., 2005). PW1+ and TWIST2+ cells are myogenic cells located within the muscle interstitium. PW1+ cells are endowed with *in vitro* and *in vivo* myogenic potential and can contribute to the satellite cell pool (Mitchell et al., 2010). TWIST2+ cells contribute mainly to type IIb/x fibers *in vivo*, and genetic ablation of these cells results in wasting of type IIb myofibers (Liu et al., 2017).

### Mesoangioblasts (MABs)

These progenitors, initially identified in the dorsal aorta of developing embryos (Minasi et al., 2002), are also present in post-natal skeletal muscle as a vessel-associated cell population. MABs have similar properties to and likely represent the *in vitro* derivative of adult pericytes (Dellavalle et al., 2007); they are capable of differentiating into several mesodermal lineages when derived from embryonic tissues, including skeletal and cardiac muscle, bone, and cartilage (Cossu and Bianco, 2003). Human skeletal muscle derived MABs are defined by the expression of the pericyte markers NG2 proteoglycan and alkaline phosphatase (ALP) and absence of endothelial markers. This enabled their prospective isolation from freshly dissociated ALP+ cells (Dellavalle et al., 2007). Their differentiation potency is restricted to skeletal and smooth muscle *in vitro* and *in vivo* (Dellavalle et al., 2011). Importantly, MABs can be genetically manipulated *in vitro* and, following intra-arterial injection, they efficiently home to skeletal muscle, contributing to regeneration in both murine and canine DMD models (Sampaolesi et al., 2003, 2006; Tedesco et al., 2011). Analysis of MAB-transplanted muscles showed that these cells also contribute, though to a limited extent, to the satellite cell pool, which represents an important aspect for ensuring long-term muscle homeostasis (Tedesco et al., 2011). Lastly, *in vitro* studies demonstrated that human MABs can also act as immunomodulatory cells, through an Indoleamine 2,3-dioxygenase (IDO) and prostaglandin E-2 (PGE)-mediated suppression of T cell proliferation (English et al., 2013).

### Bone Marrow Derived-Hematopoietic Stem Cells and CD133+ Cells

Cells endowed with myogenic potential have also been identified in the hematopoietic system (Ferrari et al., 1998). While bone marrow transplantation showed inefficient muscle regeneration (Ferrari et al., 2001), promising results were obtained using circulating CD133+ cells, also defined as CD133+ cells (Torrente et al., 2004). These cells display: (i) robust *in vitro* and *in vivo* myogenic properties; (ii) skeletal muscle homing capability upon intra-arterial delivery; (iii) ability to restore dystrophin expression; and (iv) contribution to the satellite cell pool (Torrente et al., 2004; Negroni et al., 2009). CD133+ cells can be expanded and genetically manipulated *in vitro*, thus enabling their application for autologous transplantations (Benchaouir et al., 2007). Preclinical studies in dystrophic dogs demonstrated that CD133+ cells can provide long-term muscle benefits in a large animal model and provided important data regarding the immune response toward the exon-skipped dystrophin produced by the donor cells (Sitzia et al., 2016).

### Mesenchymal Stromal Cells

Mesenchymal stromal cells (MSCs), also defined as mesenchymal stem cells, were originally identified in the bone marrow stroma and subsequently in multiple different tissues. These cells display self-renewing potential and the ability to differentiate into bone, cartilage, adipocyte and fibroblasts (Sacchetti et al., 2007; Bianco et al., 2013). Within the bone marrow, MSCs are located in a subendothelial position on the outer surface of sinusoids, a characteristic type of blood vessels (Bianco et al., 2013). In human, prospective isolation of perisinusal MSCs was demonstrated using CD146, CD105, ALP, STRO-1 and VCAM1 (Chan et al., 2009), in contrast to CD105, CD90, and VCAM1 used for their murine counterpart (Vieira et al., 2012). As there are contradictory results regarding the myogenic differentiation potential of MSCs (Gang et al., 2009; Vieira et al., 2010, 2012), it has been proposed that MSCs exert their function mainly through paracrine/secretory activity, supporting the idea that they have immune-modulatory properties (Ichim et al., 2010).

### Pluripotent Stem Cell-Derived Myogenic Progenitors

Given their ability to generate all of the cell types within an organism, pluripotent stem cells (PSCs) are an attractive potential source of myogenic progenitors for both basic and translational studies (for a comprehensive review, please see Magli and Perlingeiro, 2017). Derivation of myogenic cells from PSC cultures can be achieved using transgene-, growth factor- or teratoma-based differentiation systems (Magli et al., 2017).

Constitutive or inducible expression of the myogenic transcription factors MyoD, Pax3 or Pax7 is sufficient for driving specification of the myogenic lineage in both murine and human PSCs (Darabi et al., 2008, 2012; Albini et al., 2013; Maffioletti et al., 2015). Myogenic progenitors generated using this method display robust *in vitro* differentiation, contribute to muscle regeneration *in vivo*, and are able to replenish the satellite cell pool. Transcriptomic analysis of PAX7-inducible human PSC-derived myogenic progenitors revealed these cells are defined by the expression of CD54 (ICAM1), Syndecan 2 and Integrin α9β1 (Magli et al., 2017). Although PSC-derived progenitors display properties exhibited by embryonic cells, exposure to the *in vivo* muscle environment is sufficient to induce a molecular switch to a postnatal phenotype (Incitti et al., 2019).

Myogenic specification of PSCs can also be achieved by sequential modulation of signaling pathways controlling embryonic patterning and muscle development (Borchin et al., 2013; Chal et al., 2015; Choi et al., 2016; Xi et al., 2017; Wu et al., 2018). In general, these differentiation protocols involve the specification of a paraxial mesoderm intermediate, which is then directed toward the myogenic lineage. These cells can be prospectively isolated using a subset of surface markers (ERBB3, NGFR, CXCR4, MET, CD24, CD10) (Borchin et al., 2013; Hicks et al., 2018; Wu et al., 2018), and express the transcription factor Pax7 (Borchin et al., 2013; Chal et al., 2015; Hicks et al., 2018; Wu et al., 2018). The combination of directed differentiation and MYOD expression has also been applied to generate MABs from human PSCs (Tedesco et al., 2012). Similar to their muscle-derived counterparts, these cells display robust *in vitro* and *in vivo* myogenic properties.

Recent studies have shown that teratomas represent potential sources of muscle and other lineage-specific progenitors (Suzuki et al., 2013; Chan et al., 2018). Myogenic cells were derived from murine teratomas induced by injecting PSCs in immunodeficient mice and purified using murine satellite cell markers (Chan et al., 2018). While murine teratoma-derived myogenic cells are endowed with myogenic regenerative potential, future studies are needed in order to validate this finding in human teratomas and to assess their safety for potential clinical application.

In combination with gene delivery (Tedesco et al., 2012; Filareto et al., 2013) or gene editing technologies (Young et al., 2016), and clinically compatible purification strategies (Magli et al., 2017), PSCs provide the opportunity to produce autologous or allogeneic myogenic progenitors for regenerative medicine applications. A critical aspect for the effective application of PSC-derived cells is their safety (Andrews et al., 2017). Since, the presence of undifferentiated cells may result in tumor formation, and both PSC reprogramming and genomic engineering may result in the acquisition of chromosomal abnormalities. While these potential issues are relevant, ongoing technological advances will likely produce novel solutions that will enhance the safety of PSC-derived cells. These include strategies aimed at generating myogenic progenitors (not myocytes) through direct reprogramming from fibroblasts (Ito et al., 2017; Bar-Nur et al., 2018; Sato et al., 2019), and inclusion of suicide genes for elimination of potential donor-derived tumors (Liang et al., 2018).

### Novel Technologies That Could Improve Cell Therapy of Muscle Disorders

An important aspect to take into consideration in regenerative medicine is the potential immune response toward proteins expressed by the transplanted progenitor cell population. This becomes more relevant in the case of genetic disorders due to mutations/lack of specific genes. Current efforts aimed at minimizing this issue include the generation of cells capable of evading immune surveillance. This can be achieved by manipulating genes underlying antigen presentation (HLA I and II), or by expressing genes that repress T cell stimulation (e.g., CTLA and PD-L1) or regulate immune function (e.g., CD47 and CD200) (Lanza et al., 2019).

In addition to these various strategies aimed at generating healthy muscle fibers, there have been attempts to intervene on the muscle environment by reducing fibrosis (Cordova et al., 2018). An interesting approach for targeting specifically fibrotic tissue was documented in murine hearts using engineered T cells recognizing proteins specifically expressed in cardiac fibroblasts (Aghajanian et al., 2019). While there are no data regarding the application of these cells in the context of skeletal muscle disorders, it is possible that future therapeutic interventions in dystrophic muscles will utilize a combination of strategies aimed at both promoting muscle repair while decreasing fibrosis using chimeric antigen receptor (CAR)-T cells.

## Cell Transplantation in Duchenne Muscular Dystrophy

DMD (OMIM#310200) is a severe X-linked neuromuscular disorder with childhood onset that causes progressive muscle weakness and degeneration resulting in functional decline, loss of ambulation and early death from cardiac or respiratory failure. DMD is caused by mutations in the *DMD* gene, which encodes for one of the largest human proteins called dystrophin that links the actin cytoskeleton to the extracellular matrix through the dystrophin-glycoprotein complex (DGC) thus regulating the proper function of skeletal muscle fibers (Hoffman et al., 1987).

The lack of dystrophin leads to progressive muscle degeneration as the consequence of membrane instability, defects in maintaining calcium homeostasis, neuronal nitric oxide synthase (nNOS) activity and NO signaling. DMD is characterized by continuous cycles of muscle degeneration and regeneration, inflammation, fibrosis and oxidative stress, which lead to progressive muscle weakening and loss of muscle fibers (Carpenter and Karpati, 1979; Allen et al., 2015). The idea of cell-based therapy has therefore been one of the logical attempts to treat DMD and was pioneered in the 1990s using myoblast transplantation, as previously described in this review. The advent of stem cell research renewed the interest in cell-based therapy for DMD in the 2000s. Here we will describe the application of muscle specific stem/progenitor cells (MPSCs) and induced pluripotent stem cells (IPSCs) for the treatment of DMD.

### Myoblasts

Although the early myoblasts trials in DMD or BMD generally resulted in limited or no clinical effect, the intramuscular injection of myoblasts always resulted in localized tissue repair at the site of injection (Skuk and Tremblay, 2014). Many years of work aimed at improving protocols of transplantation (Zhu et al., 2007; Gargioli et al., 2008; Gilbert et al., 2010; Morgan et al., 2010; Liu et al., 2012) led to significant dystrophin expression in rodents, non-human primates and patients (Skuk et al., 2004, 2006), although expression remained invariably confined to the sites of injection. During the follow-up visits, which in some cases were performed more than 20 years after the procedure, no significant myoblast-related adverse effects were reported. Observed complications were mostly due to immunosuppression when this was used. This reinforced the idea that myoblast and in particular autologous myoblast transplantation could be developed as a therapeutic approach for muscular dystrophies, especially for localized forms, where intra-muscular injection may elicit a significant benefit. This will be described in relation to Oculo-Pharyngeal Muscular Dystrophy (OPMD).

### Mesoangioblasts (MABs)

MABs have been considered highly attractive for the treatment of muscular dystrophies as they can be systemically delivered and egress blood vessels to colonize the “inflamed” dystrophic muscles. Several studies have shown safety and efficacy in different animal models of muscular dystrophy, including DMD (Sampaolesi et al., 2003, 2006; Díaz-Manera et al., 2010; Tedesco et al., 2011; Domi et al., 2015). Preclinical results encouraged a phase I/IIa clinical study in humans. Allogenic intra-arterial HLA-matched donor cell transplantation in five DMD patients under immunosuppression (8–12 years; 3 ambulant and 2 non-ambulant; total infused dose 0.23 × 10^8^/Kg to 0.97 × 10^8^/Kg; EudraCT 2011-000176-33) was performed (Cossu et al., 2015). The study was relatively safe, as only one patient developed a clinically mute thalamic stroke possibly due to the angiographic procedures and/or intercurrent atrial fibrillation (1 event out of 23 total infusions). Functional measures were transiently stabilized in 2 out of 3 ambulant patients, muscle biopsy revealed some donor-derived dystrophin in one of them, but general low level of donor DNA was detected. Follow-up visits 5 years later did not reveal any further side effects or abnormal findings in these patients (Previtali, Torrente, Cossu, unpublished results). Critical re-evaluation of results suggested that low efficacy might have been due to the advanced age of the patients and consequent fibrosis, steroid therapy (reducing MABs extravasation) and insufficient number of infused cells. A new study involving intramuscular injection of autologous lentiviral-modified MABs to promote exon skipping of exon 51 is currently underway in Manchester and will confirm the level of efficiency of MABs integration and function in human skeletal muscle.

### Bone Marrow Derived-Hematopoietic Stem Cells (HSCs)

Although initial studies raised excitement regarding the possibility to treat DMD using bone marrow transplantation (Ferrari et al., 1998; Bittner et al., 1999), later reports demonstrated that HSC transplantation in mdx mice contributed to <1% of total muscle fibers, with no increase in dystrophin expression (Ferrari et al., 2001). Negative results have also been reported upon delivery of HSCs in dystrophic dogs (Dell’Agnola et al., 2004), and in DMD patients (Gussoni et al., 2002; Kang et al., 2010).

### CD133+ Cells

These multipotent stem cells have been shown to ameliorate dystrophic phenotype and dystrophin expression in mdx mice (Torrente et al., 2004). These cells have also been validated in the context of genetic correction by exon skipping strategy, suggesting their utility in the autologous transplantation setting (Benchaouir et al., 2008). More recent studies in GRMD dogs confirmed positive results in transplanted animals (Sitzia et al., 2016), while studies in humans suggest reduced regenerative capacity when these cells are harvested from DMD patients (Meng et al., 2018). Consistent with this, a clinical trial of intramuscular transplantation of CD133+ showed no integration of the donor cells in the muscle fibers (Torrente et al., 2007).

### Mesenchymal Stromal Cells (MSCs)

MSCs are endowed with immune-evasive and anti-inflammatory properties that could be useful for treating secondary processes caused by the lack of dystrophin (Ichim et al., 2010). A number of studies have investigated the role of MSCs in DMD mouse models, either by intramuscular or systemic administration, showing modest engraftment but no significant improvement of muscle contractile force nor of dystrophin expression (De Bari et al., 2003; Chan et al., 2007; Liu et al., 2007; Feng et al., 2008; Shabbir et al., 2008; Gang et al., 2009).

Human adipose-derived stromal cells (hASCs), characterized by the 12 cell surface proteins HLA-DR, HLA-ABC, CD13, CD29, CD31, CD34, CD44, CD45, CD73, CD90, CD105, and CD11, have also been reported to have some beneficial effects. hASCs were systemically administered in the DMD mouse (mdx) and dog (GRMD) models and were shown to be well-tolerated, with good muscle engraftment and some human dystrophin expression (Rodriguez et al., 2005; Vieira et al., 2012; Pelatti et al., 2016). Similarly, human pulp derived stromal cells showed significant engraftment in GRMD dogs but modest dystrophin expression after systemic administration (Kerkis et al., 2008), whereas intramuscular injection of human microfragmented fat into mdx mice exhibited anti-fibrotic and anti-inflammatory properties with minimal force improvement (Bouglé et al., 2019). More recently, adipose tissue-derived CD146+ cells, representing a pericyte subpopulation with myogenic potential, also showed some beneficial effects in double dystrophin-utrophin knock-out mice by promoting survival, but only during the treatment period, suggesting that these cells have only a short-term effect (Gomes et al., 2018).

Wharton’s jelly (WJ)-derived MSCs (human umbilical cord) showed significant anti-fibrotic and anti-necrotic activity when injected into *mdx* mice possibly through metalloproteinase-1 release (Choi et al., 2020). Umbilical cord-MSCs have also been tested in humans through the intravenous infusion of three patients with BMD (apparently 1 × 10^7^ cells). The authors reported safety, anecdotal gait improvement only in the pediatric patient and no changes in the pathology (Li et al., 2015). Intra-arterial and intramuscular administration of allogenic WJ-MSCs were subsequently investigated in nine DMD (8–14 years; 4 ambulatory and 5 non ambulatory; 16 × 10^6^ cells/kg) patients (NCT02484560). Again, the procedure was reported safe, promoted improvement in pulmonary tests, some dystrophin expression and CK reduction, but no improvement in motor outcomes (Dai et al., 2018).

Finally, recent intramuscular injection of allogenic HLA-matched bone marrow-MSCs and skeletal muscle-derived PSCs has been performed in three DMD patients (11, 22, and 12 years; 700–860 × 10^6^ cells in 9–18 cm^2^), with short-time immunosuppression (Klimczak et al., 2020). Pathology and motor functions did not change, reduction in CK levels is questionable while some anti-inflammatory effects and minimal dystrophin expression is reported.

### Pluripotent Stem Cell-Derived Myogenic Progenitors

Intramuscular transplantation of both mouse and human IPSC-derived myoblasts into *mdx* mice showed contribution to muscle formation and dystrophin expression (Mizuno et al., 2010; Darabi et al., 2012), even in the absence of a pre-treatment damage due to cardiotoxin injection (Zhao et al., 2020). Similar findings have been achieved with direct reprogramming of IPSCs into myogenic progenitors (Bar-Nur et al., 2018), even when obtained with small molecules and thus avoiding problems related to gene infection (He et al., 2020). Subsequent studies showed that myogenic progenitors derived from genetically corrected dystrophic PSCs can promote muscle regeneration when administered to *mdx* mice (Filareto et al., 2013; Young et al., 2016; Hicks et al., 2018). Similarly, genetically corrected PSCs can be differentiated into MABs, which are endowed with muscle homing potential upon systemic delivery (Tedesco et al., 2012). Further studies are necessary to assess if these cells can be used in human clinical practice due to possible risk of tumor formation. Strategies aimed at promoting the bed-to-bedside transition of PSC-derived myogenic progenitors include the identification of surface markers enabling purification and successful *in vivo* engraftment in *mdx* mice (Magli et al., 2017).

## Oculo-Pharyngeal Muscular Dystrophy

As mentioned above, myoblasts may not be the ideal candidate to treat muscle diseases which affect the majority of the body musculature, such as DMD, but they are the ideal candidate for treatment of muscle diseases affecting selectively small muscles such is the case in OPMD. In fact, while cell therapy protocols usually involve transplantation of gene-corrected autologous cells or a combination of heterologous cells and immune suppression, some muscular dystrophies with only a few affected muscles, such as OPMD, can benefit from autologous transplantation of autologous, non-modified cells from a non-affected muscle.

OPMD is an autosomal dominant genetic disease characterized by a late onset. The mutation that causes OPMD is an abnormal trinucleotide repeat expansion in the PABPN1 gene, which affects primarily a small group of specific muscles (eyelid and pharyngeal), leading to ptosis and dysphagia (Banerjee et al., 2013).

When myoblasts were isolated from the unaffected muscles of OPMD patients, they were shown to have the ability to proliferate and differentiate in a manner identical to cells isolated from normal control muscle (Périe et al., 2006). This suggested that the autologous transplantation of myoblasts isolated from the unaffected muscles of OPMD patients and transplanted into the affected pharyngeal muscles could be a possible therapy to restore muscle function and improve swallowing and quality of life. Based on these results a phase I/IIa clinical study (ClinicalTrials.gov, NCT00773227) was carried out using autologous myoblasts isolated from clinically non-affected muscles, expanded *in vitro* in GMP conditions. A median of 178 million myoblasts were injected into 12–20 different sites in the upper pharyngeal muscles of 12 patients. Improvement in the quality of life was observed in all 12 patients. The results of the trial demonstrated safety and good tolerance of the procedure with no adverse side effects. A cell dose dependent functional improvement in swallowing was even observed in this safety study. This trial supports the hypothesis that autologous myoblast transplantation into the pharyngeal muscles of OPMD patients is a safe and efficient therapeutical procedure to alleviate dysphagia (Périé et al., 2014).

## Other Muscular Dystrophies

Until now, DMD and OPMD are the only two forms of muscular dystrophy for which cell-based clinical trials have been performed. In OPMD, muscle stem cells from unaffected muscle were transplanted into diseased pharyngeal muscle and indeed improved swallowing. However, OPMD is a localized disease. Limb Girdle MDs would not profit from an autologous setting in which a population of mutated muscle stem cells is transplanted from one muscle to another. The possibility to repair mutations in muscle stem cells has become a realistic perspective due to the introduction of the CRISPR/Cas9 system into clinical medicine. Indeed, it can be shown that primary human muscle stem cells can be genetically corrected with almost 100% efficiency (Escobar et al., 2021). An autologous transplant of corrected muscle stem cells has developed into a realistic perspective. With this, all the monogenic recessive muscular dystrophies are in principle amenable to cell transplantation in patients, once the problems of delivery to many different muscles and of low engraftment will have been solved. Indeed, also dominant forms would be treatable, but the approach would require silencing of the mutated, dominant allele while guaranteeing expression of the wt allele at sufficient levels to avoid haploinsufficiency.

Congenital muscular dystrophies are severe, early onset, rapidly lethal diseases. They represent a dramatic unmet clinical need and, ethically, would be compatible with a more aggressive therapeutic approach (e.g., chemotherapy to kill diving myogenic progenitors and thus enhancing engraftment of genetically corrected cells). In these cases, there is no time to wait for future alternative strategies. It should be noted though that gene therapy for myotubular myopathy resulted in the death of two patients following AAV administration (Wilson and Flotte, 2020).

Pre-clinical evidence of efficacy in mouse models has accumulated over the last 20 years (Vilquin et al., 1999; Leriche-Guérin et al., 2002; Sampaolesi et al., 2003; Tedesco et al., 2012; Meregalli et al., 2013; Domi et al., 2015; Frattini et al., 2017; Azzag et al., 2020). Nonetheless, it should be remembered that results obtained in mice are usually superior to what later observed when the same approach is tested in large animal models (almost exclusively the dog in the case of MD) and in patients. It this thus probable that these other muscular dystrophies will have to wait the successful completion of work on DMD.

## Mitochondrial Myopathies

Skeletal muscles are high energy requiring tissues and this energy is predominantly produced by the mitochondrial oxidative phosphorylation (OXPHOS) system. Impaired OXPHOS functioning can result in a mitochondrial myopathy (MM), which is one of the most common manifestations of adult-onset mitochondrial disorders. Mitochondrial disorders are both clinical and genetically heterogeneous and often involve multiple organ systems. MM patients commonly present chronic progressive external ophthalmoplegia (CPEO), either as an isolated symptom or in combination with proximal myopathy, exercise-induced muscle pain and/or fatigue (Greaves et al., 2010; Ahmed et al., 2018). In contrast to muscular dystrophies, MM is generally slowly progressive and is not associated with severe muscle wasting (Shieh, 2013; de Barcelos et al., 2019). MM has a large impact on the patient’s quality of life and no effective treatment is currently available. Dietary supplements, aimed at improving mitochondrial function, are often prescribed, but the long-term efficacy has not been demonstrated (Pfeffer et al., 2012; Ahmed et al., 2018; de Barcelos et al., 2019).

The mitochondrial OXPHOS system is under control of both the nuclear and the mitochondrial genome. Nuclear encoded genes include subunits and assembly factors of the OXPHOS system, as well as proteins for mtDNA maintenance, and large number of mutations have been identified in >250 of the ∼1,500 nuclear encoded genes linked to OXPHOS functioning (Lightowlers et al., 2015; Alston et al., 2017). In addition, disease-causing point-mutations and large-scale deletions have been identified in the 16,569 bp circular mitochondrial DNA (mtDNA). Since each cell has multiple mtDNA copies, a mtDNA mutation can be present in all mtDNA copies, called homoplasmy, or there can be a mixture of both wild-type and mutated mtDNA copies in a cell, which is called heteroplasmy. In contrast to nDNA mutations, in which the amount of wild-type protein is either reduced by 50% or is completely absent, the effect of a heteroplasmic mtDNA mutation on mitochondrial functioning depends on the percentage of mutated copies present, ranging between 0 and 100%, and on the tissue specific threshold. In skeletal muscle of 40 MM patients with either a mtDNA point-mutation or a large-scale deletion, a negative correlation has been identified between the mtDNA mutation load and OXPHOS functioning (Taivassalo et al., 2003), and a positive relation has been shown between the skeletal muscle mtDNA mutation load and exercise intolerance in 51 m.3242A>G mutation carriers (Jeppesen et al., 2006). Increasing the total mtDNA copy number demonstrated to alleviate symptoms in mice with a pathogenic heteroplasmic mtDNA mutation encoding tRNA alanine (Filograna et al., 2019), suggesting that either a partial reduction in mtDNA mutation load or an increase in the number of mtDNA copies per cell would facilitate normal OXPHOS functioning and could potentially result in clinical improvement. In line with this, increasing mitochondrial biogenesis by endurance exercise training has been successfully applied in mtDNA mutation carriers (Taivassalo et al., 2001; Jeppesen et al., 2009), but is often not feasible because of exercise-induced lactate production or due to other clinical symptoms. Also, preferential replication of the mutated mtDNA can occur, which will worsen OXPHOS function (Taivassalo et al., 2001). In addition to endurance exercise training, a 12-week resistance-type exercise training program, aimed at inducing myogenesis, resulted in increased muscle strength, muscle regeneration, improved oxidative capacity, and increased number of NCAM+ satellite cells in carriers of a sporadic large-scale mtDNA deletion (Murphy et al., 2008). Given the fact that satellite cells of sporadic large-scale mtDNA deletion carriers have been demonstrated to contain either a low or absent level of deleted mtDNA (Moraes et al., 1989; Spendiff et al., 2013), and the observation that resistance-type exercise resulted in a small yet non-significant reduction in heteroplasmy level (Murphy et al., 2008), suggests that satellite cell activation is a valid therapeutic approach in order to induce muscle regeneration and improve mitochondrial function. Moreover, mtDNA mutation free satellite cells have been identified in carriers of a sporadic mtDNA point-mutation (Fu et al., 1996; Shoubridge et al., 1997; Taivassalo et al., 1999), and Shoubridge et al. (1997) demonstrated by repeating biopsies at the damaged muscle site, that the regenerating fibers contained solely wild-type mtDNA copies, suggesting the incorporation of satellite cells can improve muscle function in carriers of a sporadic mtDNA deletion. Since most mtDNA mutation carriers display systemic muscle weakness, satellite cells’ requirement for intra-muscular injection strongly limits their application due to requirement of the large amounts of injections (Morgan et al., 1993). Alternatively, MABs have the ability to be systemically delivered via intra-arterially transplantation. Transplantation of donor MABs has been shown to result in functional improvement of multiple muscular dystrophy animal models (Sampaolesi et al., 2003, 2006) and was found to be relatively safe in children with DMD (Cossu et al., 2015). The identification of mutation-free satellite cells in mtDNA mutation carriers, prompts the question if MABs of these patients also display low or absent mutation load. A study of 19 patients with maternally inherited mtDNA point mutations and six carriers of a sporadic large-scale mtDNA deletion, demonstrated that all of the large-scale mtDNA deletion carriers displayed wild-type MABs and nearly half of the point mutation carriers display a mtDNA mutation load below <10%. All mtDNA-mutation free MABs displayed normal mitochondrial function, demonstrating their suitability for therapeutic application (van Tienen et al., 2019). Currently, a phase I clinical study (EudraCT 2016-001258-16) is ongoing in which autologous mtDNA-mutation free MABs are intra-arterially delivered in m.3243A > G mutation carriers, which will provide insights into the safety and potential of autologous MABs cell therapy for MM. The application of autologous cell therapy would be beneficial over allogeneic stem cell therapy, as it does not require the use of immunosuppressive agents. This would be beneficial for the patient, as an immunosuppressive regimen makes them more vulnerable to infections (Porrett et al., 2014; Halter et al., 2015). Moreover, for intra-arterial MABs transplantation, inflammation is required for skeletal muscle engraftment, which might be impaired by the use of immunosuppressive agents (Cossu et al., 2015). It should be noted that MM patients generally do not display significant muscle inflammation and inflammation needs to be induced prior to intra-arterial delivery of MABs. This can be induced by a bout of maximal eccentric exercise that results in upregulation of inflammation markers, such as SDF1α and TNFα, which are associated with increased migration of MABs (Galvez et al., 2006; Valero et al., 2012; Nederveen et al., 2018).

In contrast to mtDNA mutation carriers, MM patients with a nDNA mutation will always require genetic correction in order to obtain nDNA-mutation free autologous stem cells for therapy. In recent years, CRISPR-Cas9 based genetic correction strategies are quickly emerging for numerous diseases (Sharma et al., 2021). For correction of MM-causing nDNA mutations, strategies will likely need to be tailored for each MM patient individually, as most patients present a unique mutation. This will be challenging, as it requires the development of a modular procedure for stem cell isolation and expansion, and inclusion of a tailored correction procedure. In line with this, for mtDNA-mutation carriers that do not display mtDNA-mutation free stem cells, lowering of the mtDNA mutation load needs to be achieved. To this end, degradation of the mutant mtDNA using mitochondrial-targeted TALEN or CRISPR/Cas9 (Bacman et al., 2013; Jo et al., 2015), and replication inhibition of the mutant mtDNA using peptide nucleic acids (PNAs) have been explored (Chinnery et al., 1999). However, given the multiple copies of mtDNA in all mitochondria per cell, all strategies require effective delivery of the components into the majority of the mitochondria in each cell. To this end, liposome-based carrier systems are being developed, such as the MITO-porter and nanosome systems (Yamada et al., 2008; Bae et al., 2017), but require further research before they can be applied to correct stem cells for clinical application.

In conclusion, (autologous) myogenic stem cell therapy for MM has not been applied yet, but the road to clinical application appears to be most straightforward for mtDNA mutation carriers, as mtDNA-mutation free stem cells can be directly obtained from a number of these patients. For MM patients with nDNA mutations and mtDNA mutation carriers that do not display mtDNA-mutation free myogenic stem cells, further research will be required in order to safely and effectively perform *ex vivo* genetic correction in a GMP-compliant procedure.

## Inclusion Body Myositis

The idiopathic inflammatory myopathies (IIMs) are a group of acquired autoimmune diseases where skeletal muscle inflammation is the predominant clinical feature (Oldroyd et al., 2017). Proximal limb weakness, myalgia, and fatigue are core symptoms, often with co-existing extra-muscular involvement, such as interstitial lung disease. The IIMs are rare with a prevalence of 2.4–33.8 per 100,000 (Meyer et al., 2015). Initially segregated into only polymyositis and dermatomyositis, increased heterogeneity within the IIM spectrum is evident, with immune mediated necrotizing myopathy, inclusion body myositis (IBM), anti-synthetase syndrome and overlap syndrome recognized subtypes. Each IIM subtype has well-defined clinical characteristics, histopathological findings on muscle biopsy, and serum autoantibody associations (Mariampillai et al., 2018; Betteridge et al., 2019). Most of IIMs respond to steroids and/or immune suppression and for this reason they are not of primary relevance for cell transplantation (Oldroyd et al., 2017).

IBM is the most common acquired myopathy in those aged >50 years. Compared to the other IIMs, IBM differs in terms of its pathological phenotype, where mitochondrial dysfunction and protein aggregation (e.g., amyloid, p62, TDP-43) are prominent clinical features, where focal involvement of forearm flexors and knee extensors is typical, as well as lack of response to conventional immunosuppression (Greenberg, 2019). There are no effective disease modifying treatments for IBM and as such, patients suffer from relentless accumulation of disability, including grip weakness, knee instability, falls, and dysphagia (Rose et al., 2015). This deterioration is accompanied by progressive, focally severe, atrophy and fatty replacement of skeletal muscle. Overall, IBM behaves clinically more like a late-onset muscular dystrophy rather than an inflammatory myopathy. Whilst such tissue damage can be seen in other IIM subtypes, it can usually be avoided with early and appropriately aggressive immunosuppressive therapy.

The strikingly focal muscle involvement seen in IBM means that of the IIMs is the most amenable to a cell therapy approach for treatment, rescuing tissue by targeted delivery of cells to the affected areas. For example, long finger flexor muscles could be targeted directly using an intramuscular injection technique or affected muscle groups could be targeted using a segmental intra-arterial injection of myogenic progenitors with potential for vessel wall migration, such as MABs (Sampaolesi et al., 2003). With similarity to targeted botulinum toxin injections for treating dysphagia, targeting specific diseased muscles could ameliorate important aspects of the disease (e.g., grip weakness), without requirement for systemic treatment administration and associated adverse effects (Schrey et al., 2017).

Despite the potential for myogenic cell therapy to offer a new therapeutic option for IIM, there has been very little research or progress in this area. One study has described the apparent beneficial effects of transplantation of adipose derived stem cells into a mouse model of IBM, but this has been published in abstract form only (Fabry et al., 2017). It should also be noted that whilst transplantation of such cells may have an anti-inflammatory effect, they do not have myogenic potential.

Regarding intra-arterial cell delivery, the inflammatory milieu in IBM muscle is expected to facilitate movement of MABs across the vessel wall if an intra-arterial delivery strategy is developed (Sampaolesi et al., 2003). As compared to SCs, MABs have better survival and migration capacity, and express adhesion molecules which enable them to traverse the vascular endothelium. Furthermore, MABs potently suppress human CD4 and CD8 T-cell proliferation and effector function *in vitro* (English et al., 2013). This may have relevance in terms of the populations of highly differentiated CD28null T-cells found in IBM muscle, known to be resistant to conventional immunosuppression and likely to be a key factor explain the treatment refractory nature of IBM (Greenberg et al., 2016; Pandya et al., 2016).

## Sphincter Incontinence

Sphincter muscles are other defined, functionally highly relevant muscles, well-suited for autologous transplantation.

Anal sphincter muscle anomalies result in fecal incontinence (FI) with devastating physical consequences and psychosocial dysfunction. FI causes major embarrassment and may lead to social exclusion. Population surveys suggest FI prevalence ranging from 2 to 17% (Perry et al., 2002; Kuehn, 2006; Siproudhis et al., 2006; Parés et al., 2015). However, the majority of affected persons do not consult a physician (Lam et al., 1999). In the French population for instance, 1 million people are concerned by FI, 350,000 of whom suffer from severe incontinence (more than once a week). This disease not only concerns the elderly (30% of people over 70 years living in long-term care institutions) but also significantly affects young women following delivery (Denis et al., 1992). A high frequency of postpartum FI has been reported following vaginal birth (4–38%) (LaCross et al., 2015). This frequency renders the disease a public health concern and a personal, social and economic handicap. FI still is confronted significant therapeutic management difficulties today despite the various existing treatments: drugs to regulate intestinal transit, anoperineal rehabilitation with biofeedback, surgical treatment by various techniques such as: sphincterorrhaphy or submucosal injections, sacral nerve stimulation and colostomy which is the last possible alternative.

Cell therapy by injection of autologous myoblasts would have many advantages over most alternative methods. A first pilot study of myoblast transplantation for the treatment of FI was carried out by an Austrian team who demonstrated the tolerance of the product (Frudinger et al., 2010). Rouen University Hospital then performed a randomized double-blind placebo-controlled phase II clinical trial (ClinicalTrials.gov, NCT01523522, MIAS-01, 24/24 patients included, autologous myoblasts produced by Rouen UH cell therapy facility) (Boyer et al., 2018). Results at 12 months post treatment demonstrated that injection of autologous myoblasts (AM) into the striated sphincter of patients with FI provides a clinical benefit, with a much higher benefit at 12 months in the treated arm than in the placebo arm (58 vs. 8%, *P* = 0.03). A positive response to autologous myoblast cell therapy was defined as a reduction of 30% in the Cleveland Clinic Incontinence CCI. Similar results were also found after the injection of frozen AM in patients initially in the placebo group, with a response rate of 60% (6/10) at 12 months (Boyer et al., 2018). It should be noted that at 6 months, transient not persistent placebo effect was observed.

In other strategy, Thin et al. (2013) reported similar results with sacral nerve stimulation (SNS), with response rates between 58 and 63%. Response was defined on the basis of a standardized stool diary with a reduction of more than 50% of FI episodes over a period of 3 weeks at 12 months post treatment. However, regular replacement of the pacemaker is required (approximately every 5 years) and in the long run it remains expensive.

Together these results strongly support the therapeutic efficacy and justify conducting a phase III multicenter trial: a multicenter randomized phase III clinical trial (MIAS-02, Myoblasts for Insufficient Anal Sphincter-02). The purpose of this study is to compare two surgical treatments of severe FI. The hypothesis of this “non-inferiority” trial is that injection of myoblast is clinically as effective as SNS in managing FI and could be proposed as an alternative therapy for these patients. The trial is currently being planned, with some (from PHRC, French National Research Program 2017) but non all needed funds yet secured.

In addition to FI, sphincter deficiencies comprise the urinary incontinence (UI) syndrome. UI is a widespread disease, affecting more than 200 million people worldwide (Norton and Brubaker, 2006). However, this subsumes different clinical forms and pathophysiology.

Stress urinary incontinence is the most common form of UI; the etiology involves a weakness of the urethral sphincter muscle. Animal models do not accurately mimic the distinct human conditions. Preclinical efficacy studies involving artificial damage of the rat urethral sphincter and consecutive autologous myoblast injection into the defect site indicate significant therapeutic potential (Chermansky et al., 2004). In line, muscle-derived cells have been investigated in numerous studies to regenerate the sphincter and improve continence function in stress UI (Aragón et al., 2018). The characterization of the cell products used as well as the mode of application, dosing and outcome measures differ. Yet, the feasibility of cell production, administration as well as safety are evident. While first efficacy data have appeared promising, the high importance of the rigorous, high-quality study design (control arm, blinding, stringent outcome measures) emerges. Interim analysis of a recent double-blinded, randomized, placebo-controlled design revealed a high placebo response rate evaluating the safety and efficacy of autologous muscle derived cells in stress UI (Jankowski et al., 2018). *Post-hoc* analyses suggested that the efficacy endpoint had not been defined restrictively enough.

Due to the heterogeneity of the UI patient population, trials focusing on defined disease etiologies and thus clinically homogenous patient subgroups are highly relevant to consider for the evaluation of the therapeutic potential of myoblasts. The exstrophy-epispadias complex (EEC) is an orphan, prenatal developmental disorder of the lower abdominal wall, bladder, and genitalia. EEC results from mechanical disruption or enlargement of the cloacal membrane (Ebert et al., 2009). Reconstructive surgery is the main treatment with relevant functional and cosmetic outcome. However, lifelong urinary incontinence remains a major medical problem. The incomplete development of the urethral sphincter leads to a small, circumscribed muscular defect (few mm). At the defect site muscle is replaced by vascularized and innervated connective tissue; the urothelium is intact (Canon et al., 2008). Current interventions primarily aim to establish social continence (def: dry interval of more than 3 h during the day; Chan et al., 2001). Bladder neck reconstruction (BNR) is a major surgery with the goal of forming a passive increase in bladder outlet resistance through irreversible anatomical reconstruction in the bladder neck region (Mollard and Mure, 2001). BNR results in social continence in half of the children (Shoukry et al., 2009). Seventy-five percentage of patients are lifelong dependent on catheterization or other non-physiologic urinary drainage (Maruf et al., 2020). Complications include recurrent infections, urinary stones, dilatation of the upper urinary tract, urinary retention, renal impairment, metabolic complications, increased risk of cancer, revision surgery, hospitalization, as well as wide-ranging limitations in daily life and severe psychological distress (Mouriquand et al., 2003; Surer et al., 2003; Ebert et al., 2020).

Treatments to establish physiologic continence are required. In a single-arm study autologous muscle cells were injected into the urethral sphincter defect of eight children with EEC (Kajbafzadeh et al., 2008). All boys included in the trial showed improvement of continence function after 6 months as measured by urodynamics (increased bladder capacity, leak point pressure, maximum urinary flow). In the 4-year follow-up, safety-related complications were not reported. Quality of life improved significantly. Five of the seven boys were reported to be physiologically continent, two socially continent (Elmi et al., 2011). These data reinforce the need for subsequent controlled studies involving international cooperation in this rare disease (in preparation: NCT04729582).

To conclude, the early implementation of the randomized and placebo-controlled clinical trial design is critical to investigate the clinical efficacy of autologous myoblast transplantation, particularly in rare diseases. Large-scale phase III trials are pending.

## Volumetric Muscle Loss, Sarcopenia, and Cachexia

Skeletal muscle has a remarkable capacity to regenerate upon injury; however, when muscle repair fails scar tissue replaces the damaged area with resultant functional impairment as seen in myopathies, muscle denervation, muscle ablation upon surgery but also in cachexia and sarcopenia. The rapid and focal loss of skeletal muscle with resultant functional impairment has been defined as VML (Minetto et al., 2021), while the progressive loss of muscle mass and function observed in elderly people or in patients affected by cancer or chronic diseases is referred to sarcopenia and cachexia, respectively (Cassano et al., 2009; Berardi et al., 2014). The current procedure for severe VML injuries involves muscle flap transplantation, generally autologous muscle flaps containing proper vasculature. Similar to muscle flaps, clinically relevant skeletal muscle tissue engineering constructs are developed in preclinical studies and they include vascular networks to improve cell survival deeper than a few hundred micrometers within the tissue, which is the limit of passive diffusion. Alternative strategies include the use of fibrin hydrogels in combination with muscle-derived stem cells or iPSC-derived myogenic progenitors (Matthias et al., 2018; Wu et al., 2021), which are associated with an improvement in muscle contractility in a murine VML model. In sarcopenic and cachectic conditions, dysregulated hormones, cytokines and mechanic loading facilitate protein degradation while impairing protein synthesis and affecting muscle fiber size and contractile functions. In particular, tumor necrotic factor alpha (TNFα) or interleukin 1 (IL-1) trigger their receptors TNFR1 and IL-1R, respectively and promote the translocation of NF-κB into the nucleus and the induction of gene transcription factors, including MuRF1 with the consequent muscle atrophy (Cai et al., 2004). Thus, it is not surprising that the majority of translational research studies explored pharmacological protocols to invert the balance between protein synthesis and degradation. Indeed, anti-inflammatory drugs to target pro-inflammatory cytokines elevated in plasma of patients with muscle wasting conditions have been extensively used albeit with mixed success (Furrer and Handschin, 2019).

More recently, independent groups have reported that muscle progenitor cells are impaired in muscle wasting conditions and in particular they accumulate in atrophic muscles without proceeding further toward myogenic differentiation. In this pathological muscle condition, the activation of NF-κB in myogenic progenitors maintains persistent expression of PAX7 that prevents the expression of late transcription factors necessary for myogenic maturation (He et al., 2013; Brzeszczyńska et al., 2016). Moreover, cell autonomous intrinsic changes due to aging affect the myogenic differentiation potential of human muscle progenitors and cannot be reversed when they are exposed to a young environment in xenotransplantation experiments (Rotini et al., 2018). BMPs and myostatin pathways are also implicated in the age-related muscle degeneration (Scimeca et al., 2017) and in general BMP-SMAD signaling blockade improves myogenic differentiation of muscle progenitors (Ono et al., 2011; Costamagna et al., 2016). Similar to satellite cells, MABs accumulated in cachectic muscles and when they are isolated from the atrophic muscles are still able to directly contribute to myogenesis *in vivo.* In C26 colon carcinoma-bearing mice the function of muscle stem cells can be preserved by the administration of interleukin 4 (IL-4) that acts as anabolic agent to skeletal muscle, reducing also systemic inflammation and increasing muscle protein synthesis (Costamagna et al., 2020). Commonly, IL-4 as well as IL-1 are considered cytokines promoting oncogenesis; however, IL-4 induces muscle regeneration and function. In addition, the presence of large necrotic regions enriched with type II macrophages and CD8+ lymphocytes in IL4-treated tumors suggests a relevant cytotoxic effect. Thus, the role of the IL-4 in cancer associated cachexia is currently ill defined and may provide the bases for the development of new therapeutic strategies combatting both tumor and cachexia.

Thus, it seems that muscle stem cell populations are still present in muscle wasting, however, they are unable to counteract the pathological condition, and some of them cannot complete the differentiation process despite maintaining their intrinsic myogenic potential. Although lineage tracing studies show that satellite cells fuse into myofibers in sedentary mice throughout the lifespan (Keefe et al., 2015), loss of function experiments provide evidence that lifelong genetic depletion of satellite cells in transgenic mice did not increase the severity of sarcopenia (Fry et al., 2015). In this view, genetic and/or pharmacological interventions to interfere with myogenic stem cell activation, autophagy or muscle protein turnover may offer preferential treatments for cachectic and sarcopenic muscles. In addition, an appealing solution for severe VML injuries is the use of stem cells combined with bio-scaffolds, fibrin gel among others, to promote muscular and vascular regeneration and limit fibrosis and scar tissue formation.

## Conclusion

By summarizing all the information described above, we may conclude:

1. Muscle diseases are diverse: hereditary or acquired, generalized or local. Pathological muscle weakness has enormous implications for the affected individual and for society as a whole, considering that certain diseases are very common. Major therapeutic breakthroughs have not reached clinical routine.
2. A complex and diversified methodological approach still needs to be optimized for the various myogenic progenitors to allow long term expansion *in vitro* with maintenance of self-renewal and differentiation potency.
3. Promising preclinical studies led to the early translation into clinical application and have demasked the related challenges and bottlenecks. Today, we can benefit from the insights of this pioneering work. This urges for further developments.
4. A variety of different approaches of cellular therapies are under development. In this review, we outline core strengths and limitations for different cell types. First promising clinical results not only have proved safety but also efficacy in localized muscle impairment (Kajbafzadeh et al., 2008; Périé et al., 2014; Boyer et al., 2018), point toward the indispensability of the precise identification and matching of biological and pharmacological properties of the used cell drug to the underlying disease to be treated. Emerging evidence will identify optimal therapeutic strategies, likely involving combinatorial therapy.
5. Cell therapies for muscle diseases need to be implemented and adequately funded. These gene and cell therapeutics are classified as Advanced Therapy Medicinal Products (ATMPs), a distinct class of drugs that was first legally defined in 2007/(REGULATION (EC) No 1394/2007 OF THE EUROPEAN PARLIAMENT AND OF THE COUNCIL of 13 November 2007 on advanced therapy medicinal products and amending Directive 2001/83/EC and Regulation (EC) No 726/2004). Well-established principles in classical drug development are transferable to these ATMPs only to a limited extent, due to the nature of cells and their mode of action. Recently, the EMA has invested significant effort in the standardization of distinct ATMP-related sub-aspects (European Medicines Agency, 2019) EMA/CAT/499821/2019. A muscle-specific addendum is pending. The pooling of expertise of basic scientists and clinicians from multiple disciplines, regulators, and drug manufacturers, offers the opportunity of facilitating the herewith described efforts and comparability. This will require the harmonization of the preclinical characterization of the investigational medicinal product. As an example, the standardization will mutually accelerate dose finding strategies.
6. Analysis of ATMP related clinical trials and potential later marketability highlight the importance of the early implementation of a randomized and controlled clinical trial design to investigate the clinical efficacy (Elsallab et al., 2020). In rare diseases, such as muscular dystrophies, highly innovative study designs must be implemented (Chow and Huang, 2020). A mutli-center collaboration may advance outcome measurements impacting patients’ everyday life. A lack of larger scale trials ultimately delays clinical readiness of innovation.
7. The ATMP status is associated with high upfront costs in developing the pharmaceutical manufacturing process and to produce the cell product for clinical trials. The innovative power in academic institutions for the emergence of these new therapies in predominantly rare disease, necessitates public funding of such studies as a vehicle for success and for clinical translation of these valid therapeutic options for today’s unmet medical needs.
8. We encourage the scientific, pharmaceutical and regulatory communities to join forces and work together to produce mutual guidelines for preclinical core requirements and on innovative strategies for clinical trial conduction in advancing cell therapies for muscle diseases and to strengthen the efforts for effective treatments.

## Members of the Study Group

H. Aldearee, Division of Cell Matrix Biology and Regenerative Medicine, The University of Manchester, United Kingdom; A. Bisson, Department of Immunology & Biotherapy, Rouen University Hospital, Normandy University, Inserm U1234, Rouen, France; L. Bragg, Division of Cell Matrix Biology and Regenerative Medicine, The University of Manchester, United Kingdom; V. Bridoux, Department of Digestive Surgery, Rouen Univesrity Hospital, Rouen, France; R. Duelen, Translational Cardiomyology Laboratory, Department of Development and Regeneration, KU Leuven, Leuven, Belgium; A. Farini, Unit of Neurology, Stem Cell Laboratory, Department of Patophysiology and Transplantation, Centro Dino Ferrari, Università degli Studi di Milano, Fondazione IRCCS Cà Granda Ospedale Maggiore, Policlinico, Milan, Italy; E. Gazzero, Muscle Research Unit, Experimental and Clinical Research Center, a Cooperation between Max-Delbruck-Center for Molecular Medicine in the Helmholtz Association and the Charitè Universitadmedizin Berlin, Berlin, Germany; N. Giarratana, Translational Cardiomyology Laboratory, Department of Development and Regeneration, KU Leuven, Leuven, Belgium; C. Giverne, Department of Immunology & Biotherapy, Rouen University Hospital, Normandy University, Inserm U1234, Rouen, France; L. Meggiolaro, Division of Cell Matrix Biology and Regenerative Medicine, The University of Manchester, United Kingdom; E. Negroni, Sorbonne Université, Inserm, Institut de Myologie, Centre de Recherche en Myologie, Paris, France; E. Porrello, INSpe and Division of Neuroscience, IRCCS Ospedale San Raffaele, Milan, Italy; R. Tonlorenzi, INSpe and Division of Neuroscience, IRCCS Ospedale San Raffaele, Milan, Italy; C. Villa, Unit of Neurology, Stem Cell Laboratory, Department of Patophysiology and Transplantation, Centro Dino Ferrari, Università degli Studi di Milano, Fondazione IRCCS Cà Granda Ospedale Maggiore, Policlinico, Milan, Italy; L. Yedigaryan, Translational Cardiomyology Laboratory, Department of Development and Regeneration KU Leuven, Leuven, Belgium; A. Zamboni, INSpe and Division of Neuroscience, IRCCS Ospedale San Raffaele, Milan, Italy.

## Author Contributions

All authors contributed to choice of the topics, literature search, writing, and revision of the text.

## Author Disclaimer

The views expressed in this publication are those of the authors and not necessarily those of the funding bodies.

## Conflict of Interest

The authors declare that the research was conducted in the absence of any commercial or financial relationships that could be construed as a potential conflict of interest.

## Publisher’s Note

All claims expressed in this article are solely those of the authors and do not necessarily represent those of their affiliated organizations, or those of the publisher, the editors and the reviewers. Any product that may be evaluated in this article, or claim that may be made by its manufacturer, is not guaranteed or endorsed by the publisher.
